# Monitoring of taurine dietary supplementation effect on parameters of Duroc boar ejaculate in summer season

**DOI:** 10.1371/journal.pone.0288317

**Published:** 2024-01-25

**Authors:** Magdalena Pribilova, Sylvie Skalickova, Lenka Urbankova, Daria Baholet, Pavel Nevrkla, Tomas Kopec, Petr Slama, Pavel Horky

**Affiliations:** 1 Faculty of AgriSciences, Department of Animal Nutrition and Forage Production, Mendel University in Brno, Brno, Czech Republic; 2 Faculty of AgriSciences, Department of Animal Breeding, Mendel University in Brno, Brno, Czech Republic; 3 Faculty of AgriSciences, Department of Animal Morphology, Physiology and Genetics, Mendel University in Brno, Brno, Czech Republic; Benha University, EGYPT

## Abstract

The aim of this experiment was to find out whether the taurine supplementation in daily ration had an effect on quantity or quality of Duroc boar ejaculate. The experiment duration was from June to August, when it could assumed the possible occurrence of heat stress. For the study was chosen 12 Duroc boars of approximately the same age and condition. The control group of 6 Duroc boars was fed only by basic diet and the experimental group of 6 Duroc boars was fed by the same basic diet with supplementation of 15 g taurine/boar/day. Ejaculate was collected once a week by hand glowed technique. From ejaculate parameters were monitored volume of ejaculate, sperm concentration, total amount of sperm, morphologically abnormal sperm, taurine concentration and GSH/GSSH concentration. From microscopic analysis, results were statistically significant in motility in June and July (P<0.05). In biochemical results, a significant difference (P<0.05) has been found between the experimental groups in the concentrations of taurine as well as GSH/GSSG in ejaculate which indicates the effect of heat stress on boars during the experimental period.

## Introduction

Seasonality is considered as one of major factor affecting pigs reproduction because of high sensitivity of sows and boars to seasonal changes **[1]**. The pig’s sensitivity to heat stress is caused by the lack of functional sweat glands and thick layer of subcutaneous fat tissue, which acts as an insulation **[2,3]**. Also, reduced thermal tolerance can be influenced by a genetic selection on accretion of muscle tissue, as skeletal muscle tissues generates a considerable amount of metabolic heat **[4]**. For optimal function of testicular tissue, testes are located outside the body cavity, where the temperature is lower by 2.5°C than the body core temperature **[5]**. In addition, scrotum of the boar is not pendulous which causes higher sensitivity of boar spermatozoa to thermal stress **[6]**. It has been shown the exposition of the scrotum to higher temperature, affects thespermiogenesis. These changes are demonstrated by a decrease of sperm production, reduction of their motility, and by the increase of morphologically abnormal sperm occurance **[7]**. The thermoneutral zone of boars ranges between 12–20°C **[8,9]**. Horky *et al*. observed the thermal zone of 10–17°C is the most appropriate temperature range for boars’ reproduction within to maintain low levels of free radicals in sminal fluid **[10]**. The physiological manifestation of thermal stress in boars have been observed above to 26°C **[11]**.

Oxidative stress is defined as an imbalance between physiological levels of free radicals and antioxidant defense and it’s considered to be the main cause of infertility **[7,12,13]**. Free radicals are naturally formed in the organism, and maintain several functions such as energy transfer, immune reaction, gene transcription or molecular signaling **[14,15]**. However, with excessive free radical production, main cellular functions could be damaged **[16]**. In term of reproduction system, the oxidative stress could cause abnormal morphology of sperm, decreased a total sperm count and their motility. Several strategies how to cope with the oxidative stress in boars and support antioxidant capacity of spermatozoa and testicular tissue to improve male fertility have ben described in the literature **[17,18]**. The rate of oxidative stress can be defined by ratio of level of reduced and oxidized glutathione (GSH/GSSG) in organism **[13,19]**.

Taurine is a derivate of essential amino acid cysteine and in some publications is taurine classified as an semi-essential amino acid **[20]**. Taurine is synthesized in many organic tissues through the dioxygenation of cysteine by cysteine dioxygenase into cysteine sulfinate, which is then metabolized by decarboxylation into hypotaurine that is oxidized into taurine **[21]**. Taurine could be found practically in all biological tissues. In sperm and seminal plasma, taurine is considered as the major amino acid. Its occurrence was found in Leydig cells and epithelial cells in epididymis **[22,23]**. The main role of taurine in the organism includes osmoregulaion and membrane stabilization and it acts as an antioxidant **[24]**. Although taurine is classified as antioxidant, it does not functioning as a scavenger of free radicals, because taurine’s sulfur is fully saturated and is unable to accept any more electrons **[25]**. The antioxidant role of taurine is indirect. It have been shown that taurine reduces free radical production, increases levels of enzymes involved in the antioxidant defense system **[26]** or, it inhibits a lipid peroxidation and protects cells from reactive oxygen spcies (ROS) accumulation **[27,28]**.

The aim of this study was to investigate the effect of taurine as a preventive supplement against the oxidative stress in boars during the summer season.

## Materials and methods

The study was conducted according to the guidelines of the Declaration of Helsinki, and approved by the Institutional Review Board (or Ethic Committee) of the Expert Commision for Ensuring the Welfare of Experimental Animals of Mendel University in Brno (protocol code 16OZ27083/2014-17214 and date of approval 20 May 2019).

### Animals

The experiment lasted from June to August (90 days) in the insemination station in Velké Meziříčí (Czech Republic). A total number of 12 Duroc boars with similar reproduction status, weight and age (255 ± 20 kg and 2 ± 0.3 years old) were selected for the experiment. All boars were fed by a basic feed mixture (Table 1) at the same dose 3.3 kg/boar/day. Chosen boars were divided in 2 groups (6 boars in each); control and experimental groups. In the experimental group, 15 g taurine/boar/day was added to the feed mixture 14 days before the start of the experiment as a habituation period. The daily dosage of taurine was based on the recommended dosage for humans under high stress (when oxidative stress on the organism is assumed) as there are no exact standards for taurine dosage for breeding boars **[29,30]**.

**Table 1:** Composition of feed mixture.

Composition:	wheat, barley, soya extracted meal, dried blood, calcium carbonate, dicalcium phosphate, sodium chloride, soya oil		
Analytical components	%	Additives	Amount
Dry matter	88.0	Vitamin A	8000.0 IU
Crude protein	17.3	Ferrous sulfate monohydrate	37.5 mg
Crude fibre	3.7	Calcium iodate anhydrous	0.4 mg
Crude fat	2.3	Copper sulfate pentahydrate	7.2 mg
Crude ash	5.4	Copper chelate amino acid hydrate	9.8 mg
Lysine	0.9	Manganese oxide	22.7 mg
Methionine	0.3	Manganese chelate amino acid hydrate	19.5 mg
Calcium	0.8	Zinc oxide	86.9 mg
Phosphorus	0.7	Zinc chelate amino acid hydrate	48.0 mg
Sodium	0.2	Se (sodium selenite)	0.3 mg

### Temperature and humidity assessment

**Fig 1:**
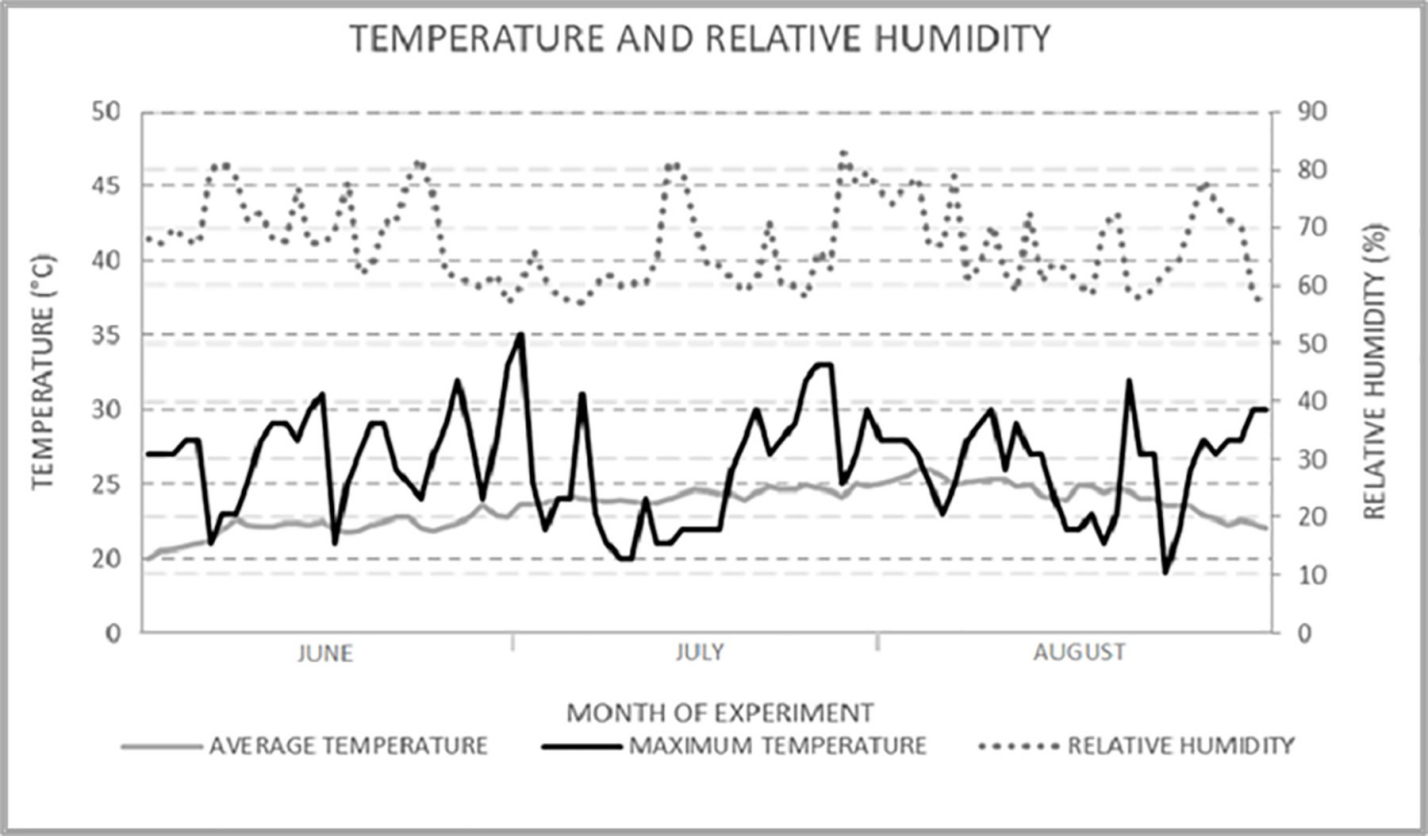
Average and maximum temperature and relative humidity measured during the experiment.

During whole experiment the temperature (°C) and relative humidity (%) were measured in the stable.Datalogger, (Voltcraft DL-121TH, Germany) was placed at the animal level (1 m above the ground). Recorded values of maximum and average temperature and relative humidity are shown in the Fig 1.

### Semen collection and evaluation

Ejaculate was taken once a week by a hand-gloved technique into tempered sterilized plastic collector. As the reproduction markers were selected parameters: volume of ejaculate (ml), concentration of sperm (10^6^/ml), total rate of sperm (10^9^/ml), motility (%), morphologically abnormal sperm (%), concentration of taurine in ejaculate (μg/ml) and concentration GSH/GSSG in ejaculate (μg/ml).

For analysis of quantitative and qualitative parameters of ejaculate was used a methodology according to Lovercamp *et al*. **[31]**:**Volume of ejaculate** was evaluated by weighing with 1 g and converted to mililiters.

**Sperm concentration** was evaluated by using a self-calibrating photometer (SpermaCueTM, Minitube of America, Verona, WI) at wavelength 340–850 nm. The sample for spectrophotometer measuring was prepared by mixing 9 mL of 1M HCl and 0.25 mL of ejaculate.

**Total sperm count** was calculated by sperm concentration × volume of ejaculate.

**Sperm motility**–the ejaculate sample (500 μL) was diluted with 500 μL of Androhep diluent and incubated in 37°C for 30 min. After incubation the sample was examined under the microscope with digital camera (Olympus microscope IX 71 S8F–3; Tokio, Japan) and evaluated using the Sperm Vision™ software (Minitube of America, Verona, WI).

**Morphologically abnormal sperm**– 50 μL of each ejaculate was fixed by 5 μl 10% buffered formalin, and than 5 μL of sample was dropped on slide, and incubated for 30 min (in 25°C and 100% humidity to immobilize the sperms). Samples were volored using congo-red and then in 0.5% aqueous solution of crystal violet. Sperm morphology was evaluated using a phase contrast microscope (Zeiss, Germany) with an oil immersion lens at a magnification of 1500×. Subjective assessment was performed by a single qualified person.

### Biochemical analysis

Prior to biochemical analysis, 800 μL of sample was immersed into liquid nitrogen for 2 minutes. After thawing, 800 Ll of MilliQ water was added to the sample, followed by vortexing for 4 minutes. The samples were placed in an ultrasonic bath (15 minutes) and then vortexed for 2 minutes. After shaking, the samples were centrifuged (25,000 rpm, 4° C, 20 minutes). Supernatant was mixed with 10% trifluoroacetic acid (1: 1) and centrifuged (25,000 rpm, 4° C, 20 minutes). After centrifugation, the collected supernatant was used for GSH, GSSG and taurine analysis.

For determination of taurine, an ion-exchange liquid chromatography (Model AAA 400, Ingos, Czech Republic) with post-column derivatization with ninhydrin and VIS detector was used. A glass column with inner diameter 3.7 mm, and 350 mm in length was filled manually with a strong cation exchanger in sodium cycle LG ANB (Ingos) with approximately 12 μm particles and 8% porosity. The glass column was tempered within the range from 35 to 95°C. A double-channel VIS detector with the volume of flow cuvette of 5 μL was set to two wavelengths– λ = 440 and 570 nm. A solution of ninhydrin (Ingos) was prepared in the mixture of 75% (v/v) methylcelosolve (Ingos) and 25% (v/v) 4 M acetate buffer (pH 5.5). Stannous chloride (SnCl2, Lachema, Czech Republic) was used as a reducing agent. The prepared solution of ninhydrin was stored under an inert atmosphere (N2) and cooled at 4°C. Elution of amino acids was performed according to program using a discontinuous gradient of elution buffers of different ionic strength and pH, and also using a temperature gradient. During the analysis, the flow rate was 0.3 mL.min-1 under the pressure of 4.5–6 MPa. Temperature was set to 120°C in the heat generator. Temperature was set to 60°C in the column **[32,33]**.

Reduced and oxidized glutathione was determined using high performance liquid chromatography with electrochemical detection (HPLC-ED). The chromatographic system consisted of two solvent delivery pumps operating in the range of 0.001–9.999 ml·min−1 (Model 582 ESA Inc., Chelmsford, MA, USA), Zorbax eclipse AAA C18 (150 × 4.6; 3.5 μm particle size; Agilent Technologies, Santa Clara, CA, USA) and a CoulArray electrochemical detector (Model 5600A, ESA). The electrochemical detector includes three flow cells (Model 6210, ESA). Each cell consists of four working carbon porous electrodes, each one with auxiliary and dry Pd/H2 reference electrodes. Both the detector and the reaction coil/column were thermostated. The sample (20 μ**L**) was injected using autosampler (Model 542 HPLC, ESA). Samples were kept in the carousel at 8°C during the analysis. The column was thermostated at 32°C. Mobile phase consisted of 80 mM TFA (A) and methanol (B). The compounds of interest were separated by the following linear gradient: 0 → 1 min (3% B), 1 → 2 min (10% B), 2 → 5 min (30% B), 5 → 6 min (98% B). Mobile phase flow rate was of 1 ml/min, working electrode potential 900 mV. Time of analysis was 20 min **[34,35]**.

### Statistical analysis

The statistical analysis was performed by STATISTIKA.CZ version 12.0 (Czech Republic). The results are expressed as the mean ± standard variance. Statistical significance was evaluated between the control and experimental group using ANOVA and Scheffe’s test–the two-factor analysis. The statistical significance was set at P < 0.05).

## Results

**Fig 2:**
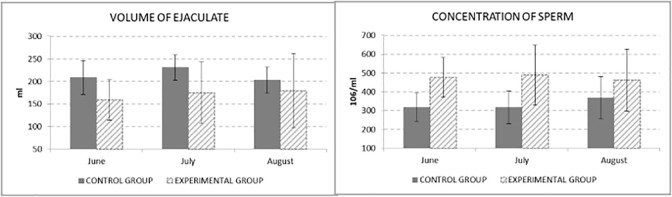
Volume of ejaculate (ml) and Concentration of sperm (10^6^/ml).

A statistically significant differences (P > 0.05) of ejaculate volume and sperm concentration were not observed between the control and experimental group in monitored months (Fig 2).

Since the concentration of sperm is negatively correlated to volume of ejaculate, the results of sperm concentration in ejaculate corresponds with results of volume of ejaculate in our experiment.

**Fig 3:**
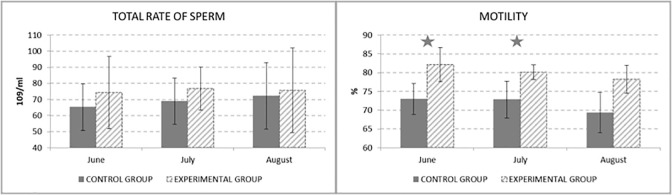
Total rate of sperm (10^9^/ml) and Motility of sperm (%).

The results of multiplying of ejaculate volume and sperm concentration could be seen in Fig 3. Experimental group showed higher level of total sperm count by 9% than control group.

From the graph on Fig 3 are obvious the statistically significant differences (P < 0.05) of sperm motility between the control and experimental group in June (by 9.15%) and July (by 7.33%). In Fig it could be seen, that in both groups there was a decrease in sperm motility during the experiment. But in control group, sperm motility decreased below the 70%, which is considered as the minimum threshold value of insemination doses.

**Fig 4:**
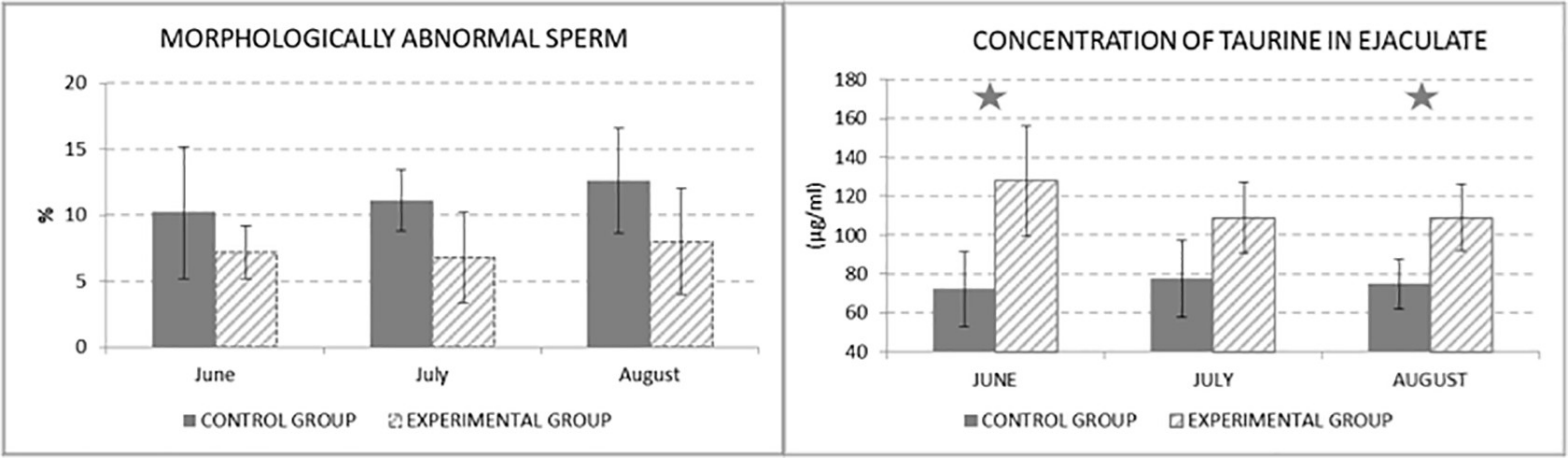
Morphologically abnormal sperm (%) and Concentration of taurine in ejaculate (μg/ml).

There were no statistically significant differences of the percentage of morphologically abnormal sperms between the control and experimental group (Fig 4), but there can be seen tendency to preserve low percentage of abnormal sperm by taurine supplementation.

Statistically significant difference between groups in taurine concentration in ejaculate could be seen in second diagram in Fig 4 in June (by 43.5%) and August (by 31.4%). According to the diagram it could be stated, that supplementing taurine in the diet caused higher level of taurine in the ejaculate.

**Fig 5:**
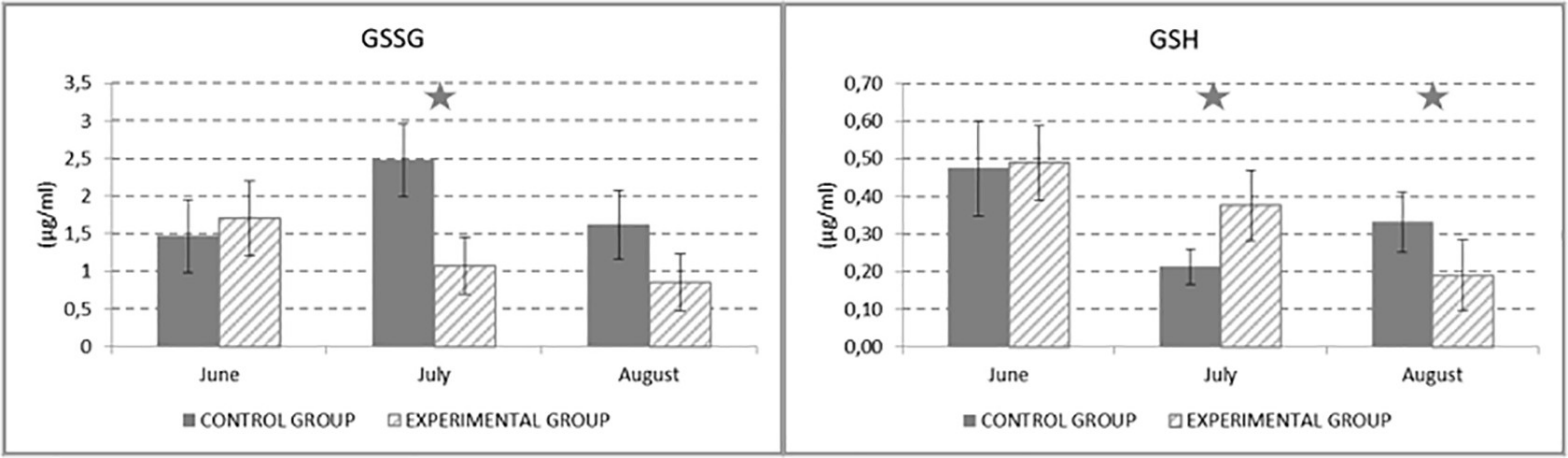
Concentration of GSSG (μg/ml) and GSH (μg/ml) in ejaculate.

The results of GSH/GSSG analysis are shown in Fig 5. In July, the statistically significant decrease (P < 0.05) of GSSG in experimental group compared to the control group is obvious. GSH concentration was significantly increased (P < 0.05) in experimental group in July and August compared to the control group. It could be expected, that in July there have been the most stressful environment in the stable, the probable oxidative stress.

## Discussion

The quality of boars’ ejaculate is mostly influenced by a season, where the best results of quality are recorded in spring and autumn months and, on the other hand, the worst quality can be observed in summer months **[36]**. For this reason, the experiment was carried out in summer season when the heat stress could be expected. From the monitoring the temperature during the experimental period it could be seen, that daily temperatures fluctuated around 26°C, which is considered as for the temperature causing te heat stress in pigs **[11]**. Patience *et al*. established the thermoneutral zone in pigs even in the range of 21–23°C **[8]**. Moreover, high temperature above the thermoneutral zone can cause oxidative stress, which is responsible for degraded quality of ejaculate **[10]**. The oxidative stress could be confirmed by several diagnostics methods. Some studies have shown a good correlation between the oxidative stress level and GSH:GSSG ratio. Reduced levels of GSH:GSSG ratio means non free radical oxidative stress–stress is not caused by lipid peroxidation or protein damage but the redox imbalance **[37]**. From our results, we can confirm, that in boars was determined redox imbalance and potential oxidative stress.

Determine an adequate dose of taurine to breeding board during heat stress is difficult, as the topic has not yet been fully researched. We had to draw inspiration in humane medicine, where recommended daily dose (in stress situations) is ranged between 1–6 g/day **[29,30]**. Since the referent human is about 80 kg, it is necessary to recalculate the daily dose of taurine for boars according to their actual weight, so the dose 15 g/boar/day had been established. In recent study, where taurine was supplemented to weaned piglets to prevent muscle damage, similar dosages were determined **[38]**.

The main function of taurine in the organism, is the oxidative stress regulation **[39]**. Taurine is involved in important biochemical reactions: protection of cell membranes, lowering toxicity of some substances or regulation of osmotic pressure in cells **[40,41]**. In sperm, taurine enhances its motility and increasing the fertilization potential of sperm cells **[42]**. From our results we can confirm, that in experimental group values of sperm motility were significantly higher when compared to the control group. Improvement of sperm motility and viability was also confirmed in other study **[43]**. The proposed mechanism of action is that taurine is present in mitochondria, which is considered as the responsible organelle for sperm movement **[44]**. Although the other parameters of boar ejaculate were not statistically significant, there could be observed a tendency to improve reproduction parameters of boar ejaculate.

In recent studies taurine was used improving a viability of sperm after short-term storage or during the cryopreservation of ejaculate of various species **[45–48]**. There’s only a few studies which investigated the effects of taurine in vivo. For example FangFang *et al*. also reported a higher total sperm count and motility and a decrease of abnormal sperm in Yorkshire boars after addition of 6 g/kg diet **[49]**. Positive influence of taurine was also proved in rats in parameters motility and total sperm count **[20]**. Since the recent research is focused mainly on in vitro supplementation of taurine into ejaculate, we cannot compare our results many other studies.
